# Evaluation of different egg quality traits and interpretation of their mode of inheritance in White Leghorns

**DOI:** 10.14202/vetworld.2015.449-452

**Published:** 2015-04-07

**Authors:** Pradeepta Kumar Rath, Prasanna Kumar Mishra, Bandi Kumar Mallick, Nrusingha Charan Behura

**Affiliations:** 1 P.G. Department of Poultry Science, College of Veterinary Science and Animal Husbandry, Orissa University of Agriculture and Technology, Bhubaneswar – 751003, Odisha, India; 2 Central Poultry Development Organisation (CPDO), Eastern Region, Bhubaneswar, Odisha, India

**Keywords:** egg quality, heritability, means, white leghorn

## Abstract

**Aim:**

The experiment was carried out to evaluate different external and internal egg quality traits and to figure out their mode of inheritance from a set of hierarchically classified data.

**Materials and Methods:**

The data collected from 548 progenies (1 egg from each progeny) of 282 dams mated to 47 sires (1 sire mated to 6 dams) of a White Leghorn flock were used in the present study. Phenotypic means and their standard errors were calculated for all the quality traits. Heritabilities were estimated for these traits separately from “sire,” “dam” and “sire+dam” (full-sib) components of variance using Statistical Package for Social Sciences-17 program.

**Results:**

External quality traits such as; egg weight, length, width, shape index, surface area, shell weight, shell thickness, shell ratio, and internal quality traits such as; length, width, height, and weight of albumen and yolk, albumen index, albumen ratio, Haugh unit (H.U.), yolk diameter, yolk index, yolk ratio, and yolk albumen ratio were measured in 548 eggs of the experimental White Leghorn flock. The eggs were found to have optimum weight (57.78±0.20 g), shape index (73.53±0.18) and shell characteristics (6 g, 0.32 mm) as per its genetic potential. Higher values for albumen height (8.41±0.04) mm and H.U. (92.00±0.19) are attributable to the freshness of eggs and proper age of hens. Heritability estimates from “sire” component of variance were higher than “dam” and “sire+dam” components for the traits like egg weight, length, width, shape index, surface area, albumen width, albumen index, H.U., yolk width, yolk height, yolk weight, and yolk index whereas for rest of the traits the values estimated from “dam” component were higher. Estimates from “sire+dam” component were intermediate to the estimates derived from “sire” and “dam” components.

**Conclusion:**

The heritability estimates from different egg quality traits were moderate to high. Since most of the traits have high heritability values, these traits can be improved by mass selection.

## Introduction

Poultry farming is one of the fastest growing segments of the agricultural sector in India today. It carries a pivotal position in current Indian economy and has evolved from subsistence farming to an extremely business oriented enterprise [1]. This transformation owes to huge investment in breeding, hatching and overall management practices by both government and private organizations. The poultry sector in India has shown a substantial improvement over the years. The total egg production reached 66.45 billion with per capita availability of 55 eggs per annum in 2011-12 and the poultry meat production was estimated to be about 2.47 million tonnes for the same year [2].

As far as egg consumption is concerned, it has been accepted worldwide as a staple food and included as an important ingredient in a balanced human diet. The quality traits of an egg are those that affect its acceptability to the consumer. Hence, to maintain superiority in the overall quality of an egg, continuous genetic evaluation of different egg quality traits has become essential in today’s production oriented market [1].

Therefore, this investigation was carried out to estimate different egg quality parameters and to understand their inheritance pattern in a white leghorn population maintained at Central Poultry Development Organization (Eastern Region), Bhubaneswar.

## Materials and Methods

### Ethical approval

All the procedures have been conducted in accordance with the guidelines laid down by the Institutional Ethics Committee.

### Experimental birds and their management

The data were collected from 548 progenies (1 egg from each progeny) of 282 dams mated to 47 sires (each sire mated to 6 dams) in a White Leghorn strain flock of Central Poultry Development Organization (CPDO), Eastern Region, Government of India, Bhubaneswar. The eggs were obtained at 40 weeks of age and data recorded on the same day of collection. The chicks, grower and layer birds were reared in floor pens and fed conventional starter, grower and layer rations respectively. A lighting schedule of 16 h/day was applied during laying period. Standard procedure with respect to preventive vaccination and medication were followed during the study period. The whole research work was conducted over a period of 1 year from July, 2013 to July, 2014.

### Measurement of external characters

The individual eggs were weighed on a digital balance to the nearest of 0.01 g accuracy. The egg length and breadth of eggs were measured with the help of digital calipers and shape index was calculated as the ratio of breadth to length times 100. Surface area (S) was calculated with a formula of S=4πr². Radius (r) was calculated as ¼ (length + breadth) of the egg. The inner shell membrane was removed from the shells and kept to dry in the open air for 24 h. All the dried shells were weighed with the help of a digital balance. The shell weight was divided by the egg weight to get the shell ratio. The thickness of four pieces of shells, one each from the two ends (broad and narrow end) and two from the body of the eggs were measured to the nearest of 0.01 mm with the help of screw gauze and averaged.

### Measurement of internal characters

The length and width of the albumen and yolk were measured in mm with the help of a vernier caliper. The height of the albumen and yolk were measured at the top by spherometer on a table glass. Care was taken to balance the table and table glass with the help of dumpy level/theodolite. The height of the albumen was measured at 3 or 4 locations and averaged. Haugh unit (H.U.) was calculated by using the formula H.U.=100 log (H+7.57−1.7W^37^) Where, H is albumen height in millimeters, measured by spherometer and W is observed weight of the egg in grams [3]. Albumen and yolk indices were estimated in percentage, taking the ratio of their respective heights to the average of breadth and length as suggested by previous workers [4]. Albumen weight was calculated as egg weight- (Yolk weight + shell weight). Albumen and yolk ratios were calculated taking their individual weights as the percentage of total egg weight. Yolk diameter was estimated as the average of yolk length and breadth. Yolk albumen ratio was calculated as weight of yolk/weight of albumen.

### Statistical analysis

Phenotypic means with standard errors were calculated for all the quality traits and heritabilities were estimated separately from “sire,” “dam”, and “sire+dam” (full-sib) components of variance using Statistical Package for Social Science-17 program.

## Results and Discussion

### Phenotypic means

Calculated phenotypic means with standard errors for different external and internal egg quality traits are presented in Table-1. The phenotypic parameters such as size and shape of eggs, eggshell thickness, characteristics of albumen and yolk were elucidated. Eggs of this flock (57.78±0.20 g) were in between the range of “large” and “extra-large” categories as mentioned by Zeidler [5]. Higher and lower means for egg weight were also reported by the earlier authors [1,6-8]. Naked neck chickens laid heavier eggs with higher mean length and width than the present white leghorn strain at 40 weeks of age [9]. There were also reports of lower mean values in comparison to the present findings for egg length (54.39±0.11 mm) and width (39.92±0.07 mm) [7]. The shape index reported under the study (73.53±0.18) falls within the “normal” range of 72-76 [10] and is lower than the earlier reports [8,9,11]. Normal eggs fit well to the trays and make less transit loss. Nonga *et al*. [6] found eggs with thicker shells to that of the experimental flock (0.32 mm) in free rage local chickens whereas thinner shells were reported in laying hens of L-line [12]. An egg shell thickness of at least 0.33 mm has been estimated to be necessary for the eggs to have at least a 50% chance to withstand normal handling condition without breakage [13]. Estimated shell weight (6 g) and shell ratio (10.42%) are higher than the previous reports in different strains of white leghorns [1]. Similarly, mean values for albumen height (8.41±0.04 mm), yolk height (18.22±0.03 mm) and yolk width (44.72±0.11 mm) estimated in the experiment are higher than the values from different genetic groups of naked neck chicken, whereas both the strains agree in their mean for albumen width [9]. Albumen height (8.41±0.04 mm) observed in the experiment is within the range value for superior quality as mentioned by Zeidler [5]. The higher albumen height may be due to the freshness of eggs and young age of hens. A higher H.U. than previous reports [1,14] corroborates the fact. Albumen index (9.98±0.05%) estimated in the flock is in close agreement to the value found in Colombian chicken [8]. Divergent findings with lower and higher means for albumen index and yolk index (40.24±0.10%) were also observed [8,11,14]. RIR chickens are observed to have heavier albumens whereas Sonali chickens are reported to have heavier yolks than the white leghorn flock under the study [15]. Albumen ratio (61.92±0.21%) and yolk ratio (28.09±0.10%) are lower than the earlier estimates [14] that means albumen and yolk contributes less to the total egg weight.

**Table-1:** Mean and standard errors for different traits.

Traits under study	Mean±SE (n*=548)
External characters	
Egg weight (g)	57.78±0.20
Egg length (mm)	54.39±0.11
Egg width (mm)	39.92±0.07
Shape index (%)	73.53±0.18
Egg surface area (cm^2^)	69.90±0.16
Shell weight (g)	6.00±0.03
Shell thickness (mm)	0.32±0.00
Shell ratio (%)	10.42±0.06
Internal characters	
Albumen length (mm)	92.37±0.26
Albumen width (mm)	76.91±0.24
Albumen height (mm)	8.41±0.04
Albumen weight (g)	35.76±0.15
Albumen index (%)	9.98±0.05
Albumen ratio (%)	61.92±0.21
Haugh unit	92.00±0.19
Yolk length (mm)	45.98±0.01
Yolk width (mm)	44.72±0.11
Yolk diameter (mm)	45.35±0.09
Yolk height (mm)	18.22±0.03
Yolk weight (g)	16.17±0.05
Yolk index (%)	40.24±0.10
Yolk ratio (%)	28.09±0.10
Yolk albumen ratio (%)	45.21±0.11

* n the total number of eggs used in the study

### Heritability

The heritability estimates of egg quality traits calculated from “sire,” “dam”, and “sire+dam” components of variance utilizing the variance components of the analysis are presented in Table-2.

**Table-2:** Heritability estimates of various traits from sire, dam, and “sire+dam” components of variance and their standard errors.

Traits	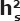	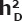	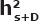
Egg weight (g)	0.443±0.160	0.277±0.232	0.360±0.131
Egg length (mm)	0.679±0.202	0.231±0.217	0.455±0.140
Egg width (mm)	0.868±0.239	0.332±0.205	0.601±0.150
Shape index (%)	0.860±0.232	0.007±0.204	0.433±0.148
Egg surface area	0.799±0.223	0.178±0.208	0.488±0.145
Shell weight (g)	0.058±0.091	0.223±0.257	0.141±0.124
Shell thickness (mm)	0.149±0.116	0.743±0.256	0.446±0.126
Shell ratio (%)	0.101±0.104	0.579±0.257	0.340±0.124
Albumen length (mm)	0.337±0.150	0.747±0.244	0.542±0.130
Albumen width (mm)	0.895±0.250	0.671±0.207	0.783±0.154
Albumen height (mm)	0.436±0.164	0.584±0.236	0.510±0.132
Albumen weight (g)	0.460±0.171	0.685±0.235	0.572±0.134
Albumen index (%)	0.528±0.176	0.273±0.227	0.401±0.134
Albumen ratio (%)	0.220±0.119	0.234±0.247	0.227±0.126
Haugh unit	0.643±0.194	0.144±0.218	0.394±0.138
Yolk length (mm)	0.114±0.105	0.335±0.254	0.225±0.124
Yolk width (mm)	0.201±0.115	0.184±0.247	0.193±0.125
Yolk diameter (mm)	0.295±0.141	0.694±0.246	0.494±0.129
Yolk height (mm)	0.751±0.225	0.736±0.218	0.743±0.147
Yolk weight (g)	0.740±0.217	0.413±0.214	0.577±0.144
Yolk index (%)	0.807±0.228	0.374±0.209	0.590±0.147
Yolk ratio (%)	0.275±0.141	0.921±0.250	0.598±0.129

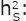
 Heritability from sire component of variance, 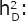 Heritability from dam component of variance, 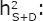 Heritability from “sire+dam” component of variance

Most of the traits were moderate to highly heritable. Egg weight was found to be moderately heritable and estimates from the “sire+dam” (0.360±0.131) and “sire” (0.443±0.160) components of variance were well comparable to the observations of earlier authors from the respective components [16,17]. The higher and lower estimates were also reported by previous workers [1,18]. The higher estimate from the “sire” component of variance might be due to sex-linked fraternal effect. Similarly, the heritability estimate for shape index from the “sire+dam” component is in agreement to the earlier report [17]. Heritability estimates from the “dam” component for the traits like egg length (0.231±0.217), width (0.332±0.205), shape index (0.007±0.204), and egg surface area (0.178±0.208) were significantly lower than estimates from “sire” component indicating less importance of maternal influence and non-additive gene action for these traits. The higher estimates of sire component for these traits indicate, there is major genetic variance between the males used in the study. For shell weight, heritability is lower to the previous estimation in White leghorn birds [19]. Whereas for shell thickness the heritability from combined component (0.446±0.126) was higher than the earlier reports in different strains of White Leghorns [1]. In the present study, heritability estimates for shell weight, shell thickness and shell ratio from “dam” component of variance were found to be higher than from “sire” component indicating the importance of non-additive gene action and maternal effect for these traits. Devi and Reddy [20] found a lower heritability (0.13-0.22) for H.U. from “dam” component of variance, which is in agreement to the current estimate from the respective component (0.144). Reported heritability value for albumen index in IWD and IWK strains by full-sib method are well comparable to the present estimate (0.401±0.134) [20], however a report of very low heritability (0.01) for this trait was also depicted in White Leghorns [21]. The moderate heritability value from “sire+dam” component indicates a “sexual dimorphism” and a scope for improvement in this trait through selection. The quality parameters of yolk (except yolk length and width) are highly heritable suggesting applicability of individual selection for these traits.

## Conclusion

The heritability estimates from different egg quality traits were moderate to high. Since most of the traits have high heritability values, these traits can be improved by mass selection. However, further studies are required to assess the genotypic and phenotypic association between different external and internal traits which will help in determining the best selection program for production of eggs with optimum quality.

## Authors’ Contributions

The present study is a thesis part of M.V.Sc degree of PKR. PKM planned the study and PKR done the research under the guidance of PKM and BKM. NCB guided in statistical analysis. All authors participated in draft and revision of the manuscript. All authors read and approved the final manuscript.
